# An *in vitro* 3D bone metastasis model by using a human bone tissue culture and human sex-related cancer cells

**DOI:** 10.18632/oncotarget.12763

**Published:** 2016-10-19

**Authors:** Francesca Salamanna, Veronica Borsari, Silvia Brogini, Gianluca Giavaresi, Annapaola Parrilli, Simona Cepollaro, Matteo Cadossi, Lucia Martini, Antonio Mazzotti, Milena Fini

**Affiliations:** ^1^ Laboratory of Biocompatibility, Technological Innovation and Advanced Therapy, Rizzoli RIT, Rizzoli Orthopedic Institute, Bologna, Italy; ^2^ Laboratory of Preclinical and Surgical Studies, Rizzoli Orthopaedic Institute, Bologna, Italy; ^3^ Laboratory of Tissue Engineering-Innovative Technology Platforms for Tissue Engineering, Rizzoli Orthopedic Institute, Palermo, Italy; ^4^ Department of Medical and Surgical Sciences, University of Bologna, Bologna, Italy; ^5^ I Orthopaedics and Trauma Clinic, Rizzoli Orthopaedic Institute, Bologna, Italy; ^6^ University of Bologna, Bologna, Italy

**Keywords:** 3D model, cancer-bone metastasis model, human bone tissue, human sex-related cancer cells

## Abstract

One of the main limitations, when studying cancer-bone metastasis, is the complex nature of the native bone environment and the lack of reliable, simple, inexpensive models that closely mimic the biological processes occurring in patients and allowing the correct translation of results. To enhance the understanding of the mechanisms underlying human bone metastases and in order to find new therapies, we developed an *in vitro* three-dimensional (3D) cancer-bone metastasis model by culturing human breast or prostate cancer cells with human bone tissue isolated from female and male patients, respectively. Bone tissue discarded from total hip replacement surgery was cultured in a rolling apparatus system in a normoxic or hypoxic environment. Gene expression profile, protein levels, histological, immunohistochemical and four-dimensional (4D) micro-CT analyses showed a noticeable specificity of breast and prostate cancer cells for bone colonization and ingrowth, thus highlighting the species-specific and sex-specific osteotropism and the need to widen the current knowledge on cancer-bone metastasis spread in human bone tissues. The results of this study support the application of this model in preclinical studies on bone metastases and also follow the 3R principles, the guiding principles, aimed at replacing/reducing/refining (3R) animal use and their suffering for scientific purposes.

## INTRODUCTION

Bone metastasis is a common finding in the natural history of several types of cancers and contributes extensively to morbidity and mortality in cancer patients [1–2]. After the identification and emergence of the ‘seed and soil’ [3] and the ‘vicious cycle’ concepts [4] numerous studies have been performed to understand the complex interaction between metastatic tumor cells and bone. The tumor cells that metastasize in the bone induce destructive osteolytic and/or bone forming osteoblastic lesions [4] and ‘teach’ this affected bone microenvironment to produce factors that stimulate tumor cell growth [5–6]. To understand the cellular and molecular mechanisms involved in cancer-bone interaction and metastasis treatment, reliable models are required to mimic the biological processes that occur in patients.

To date traditional cancer bone metastasis models (*in vitro* and *in vivo*) contain inherent limitations concerning controllability, reproducibility and flexibility of design. Conventional 2D cell culture models have provided a better understanding of the ability of tumor cells to grow, but they do not provide information about the complex interactions between the cancer cells and the physicochemical environment that exists within living tumors [7]. Such limitations may offer less reliable data thus leading to restrictions for translation into clinical application. *In vivo* animal models are more suitable and overcome many of the limitations of the 2D model, but their inability to accurately mimic human cancer-bone metastasis, combined with the need to study metastases under controlled conditions, has led to the emergence of alternative *in vitro* models based on 3D cell cultures. This new field of research lies at the intersection between pathology and tissue engineering by providing an important alternative to both complex *in vivo* whole organism approaches, and 2D culture with its spatial limitations [8]. Since advances in culture techniques have emerged from the field of tissue engineering, 3D cell culture methods have increased greatly in number [9–10]. In fact, biomaterials such as natural or synthetic matrices/scaffold (e.g. Poly (DL-lactic-co-glycolic) acid, Poly (Lactide-co-Glycolide), chitosan, alginate, collagen) ordinarily employed for engineering bone and cartilage tissue [11–14] have also been used for culturing cancer cells [15–6].

However, one of the main physiological approaches may be the culture of bone tissue explants that allow the 3D architecture, the tissue extracellular matrix and the cellular complexity to be preserved *in vitro*.

Nordstrand et al. implemented a murine calvarial explant to monitor how tumor cells influenced the bone remodeling process and how the bone microenvironment influenced the tumor cells [17]. To achieve this, they established a two-compartment *in vitro* co-culture and used it to follow the tumor-induced bone remodeling. Similarly, Schiller et al. developed a model where whole neonatal mouse femurs were co-cultured with a variety of mouse and human cancer cell lines and found bone tissue viability and described the synergistic paracrine interactions between intact bone and tumor [18]. Others studies have cultured mouse bones harvested from breast cancer xenograft models to examine the cytokine production of mouse bones colonized by breast cancer cells. These studies showed the feasibility of bone co-culture models to stimulate the microenvironment and studied the dynamic cell interactions within the bone metastatic niche [19–20]. Furthermore, several studies of tumor biology have benefited from the hypoxia setting which mimics the nutrient and oxygen insufficiency at the tumor-host interaction [21]. Based on these findings, Curtin et al. [22] utilized a 3D cancer-bone metastasis model composed of free-floating live mouse calvarial bone organs in the presence of cancer cells in a roller tube system under hypoxic conditions. The study developed a 3D model which simulates closely the *in vivo* tissue under defined conditions. These 3D *in vitro* models, where human cells are cultured together with animal tissues, well mimic the *in vivo* condition, but leave out the considerable aspect of species-specific osteotropism. In fact, several authors [23–24] highlighted that human breast cancer cells preferentially home to human bone fragments implanted in mice, thus underlining the species-specific behavior. Nevertheless, to the present authors' knowledge there is a lack of 3D models with the use of human bone. The use of human bone and human cells would allow patient factors that influence the development of bone metastases to be studied, i.e. age, sex, concomitant comorbidity and unhealthy life style factors. Recently, Contag et al. [25] developed a co-culture model able to monitor dynamic interactions between human breast cancer cells and human bone tissue. The authors used a static model where the bone marrow was depleted from the bone tissue and cancer cell/bone interactions were evaluated during relatively short time periods (for up to 96 hours) and not in hypoxic conditions, which is typical of tumors grown *in vivo*.

For all these reasons, and taking into account the 3R principle aimed at reducing the use of laboratory animals and their suffering, a 3D bone metastasis model was developed. The *in vitro* 3D cancer-bone metastasis model was performed by culturing human breast or prostate cancer cells with discarded human bone tissue isolated from the femoral head after total hip replacement surgery, from female and male patients respectively. Hypoxia was also applied to the culture to reflect the *in vivo* conditions able to simulate the nutrient and oxygen insufficiency in tumor-host interaction. This study allowed to set-up an *in vitro* 3D sex-related bone metastasis model, able to recapitulate the different stages of breast and prostate cancer bone metastasis. This interactive system, through histological, immunohistochemical and 4D micro-CT analyses and by investigating specific markers predictive of bone remodeling, appears to be appropriate for the study of bone metastases.

## RESULTS

### Bone tissue viability

**Figure 1:**
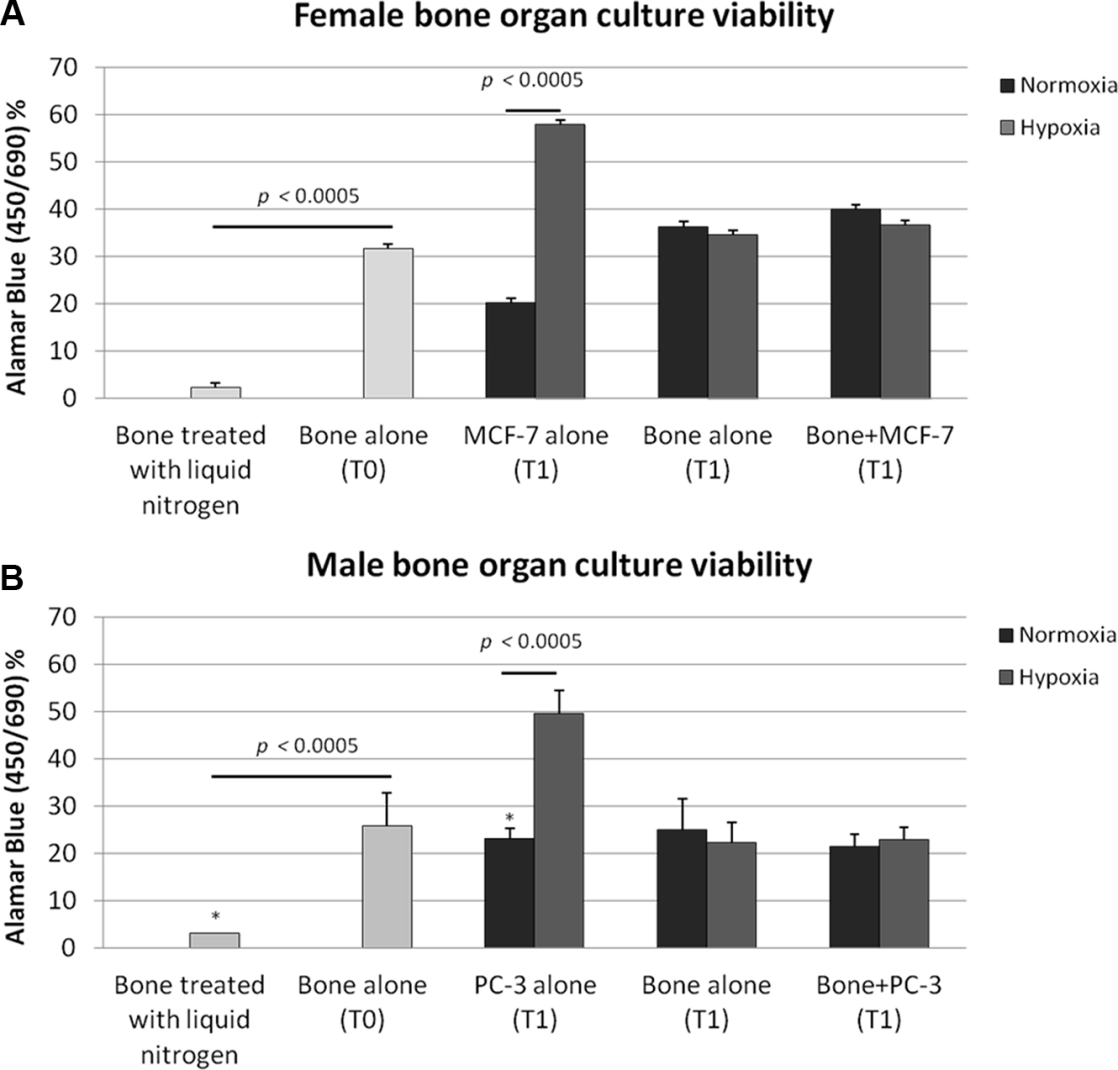
Bone tissue culture viability in (**A**) female and (**B**) male bone specimens treated with liquid nitrogen (necrotic bone, T0), in bone specimens alone (T0), in (A) MCF-7 and (B) PC-3 cultured alone in the TubeSpin Bioreactors in hypoxic and normoxic conditions (T1), in femoral head specimens alone (T1), in femoral head specimen co-cultured with 2.5 × 10^4^ cancer cells in both hypoxic and in normoxic condition (T1). *T-test* analysis: *p< 0.0005.

We studied cancer/cell bone interaction for relatively long periods for up 7 days, and the alamar blue test confirmed the bone organ viability (Figure 1A and 1B). At T0 both female and male femoral head specimens showed a high level of bone viability (Figure 1A and 1B). Bone viability was significantly higher at T0 when femoral head specimens were compared to those whose bone marrow was depleted through the liquid nitrogen treatment (*p <* 0.0005) (Figure 1A and 1B). The bone viability was also maintained after 7 days of culture in the TubeSpin Bioreactors on the rolling tube apparatus with and without cancer cells (MCF-7 and PC-3 respectively) and in both normoxic and hypoxic conditions without significant differences. When MCF-7 or PC-3 were cultured alone in the TubeSpin Bioreactors on the rolling tube apparatus significant differences between normoxic and hypoxic conditions, in both female and male femoral head specimens, were observed. In fact, as shown in Figure 1A and 1B both MCF-7 and PC-3 revealed significantly higher values in hypoxic conditions in comparison to normoxic ones (*p <* 0.0005).

### 4D micro-CT analyses

**Figure 2:**
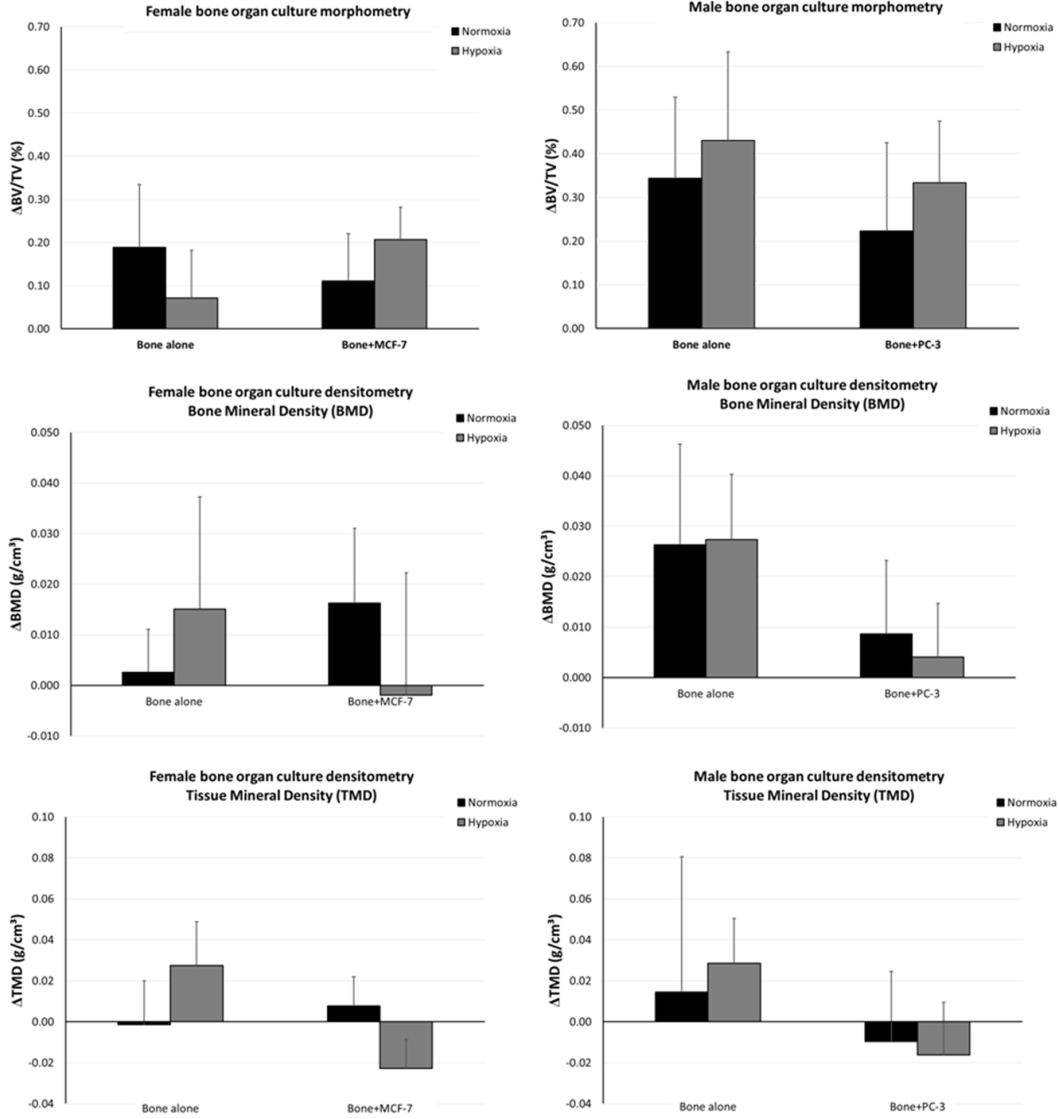
Changes over time in BV/TV and in BMD and TMD in response to 3D *in vitro* tumor cell culture

The changes over time in bone volume fraction (BV/TV) and mineral density (BMD and TMD) parameters in response to 3D *in vitro* tumor cell culture is shown in terms of differences between the two experimental times (time 0 and 7 days) (Figure 2). No significant differences were observed among groups.

### ELISA protein assay

**Figure 3:**
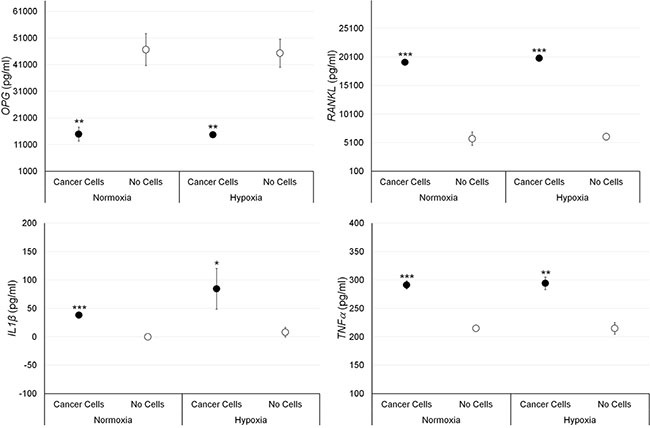
Protein releases (ELISA), expressed as total protein ratio (*n* = 3 replicates), in female bone fragments cultured with or without MCF-7 cancer cells, both in normoxic and hypoxic conditions RANKL (normoxia and hypoxia ***p < 0.0005), IL-1β (normoxia: ***p < 0.0005; hypoxia: *p < 0.05) and TNF-α (normoxia: ***p < 0.0005; hypoxia: **p < 0.005): bone fragments with MCF-7 *versus* bone fragments without cancer cells. OPG (normoxia and hypoxia **p < 0.005): bone fragments without cancer cells *versus* bone fragments with MCF-7.

**Figure 4:**
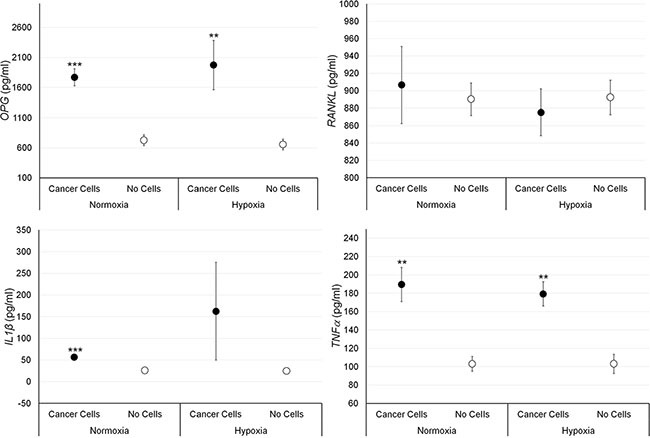
Protein releases (ELISA), expressed as total protein ratio (*n* = 3 replicates), in male bone fragments cultured with or without PC-3 cancer cells, both in normoxic and hypoxic conditions OPG (normoxia: ***p < 0.0005; hypoxia: **p < 0.005), TNF-α (normoxia and hypoxia: **p < 0.005), IL-1β (normoxia: ***p < 0.0005): bone fragments with PC-3 *versus* bone fragments without cancer cells.

The effect of MCF-7 and PC3 cells on bone fragments was investigated through evaluation of OPG, RANKL, IL-1β and TNF-α expression. Results of significant data are summarized in the graphs reported in Figures 3 and 4. RANKL and TNF-α production was significantly increased in bone fragments cultured with MCF-7 breast cancer cells in comparison to bone fragments cultured without cancer cells, both in normoxic and hypoxic conditions (Figure 3). Conversely, OPG expression was significantly reduced in bone fragments cultured with MCF-7 cells compared to bone fragments cultured without cancer cells, both in normoxic and hypoxic conditions (Figure 3).

Bone fragments cultured with PC-3 cells showed significantly higher values of OPG and TNF-α expression compared to bone fragments cultured without prostate cancer cells, both in normoxic and hypoxic conditions, while IL-1β expression was significantly higher, only under normoxic condition (Figure 4). No significant differences were found regarding RANKL expression in presence of prostate cancer cells.

### Gene expression analyses

**Figure 5:**
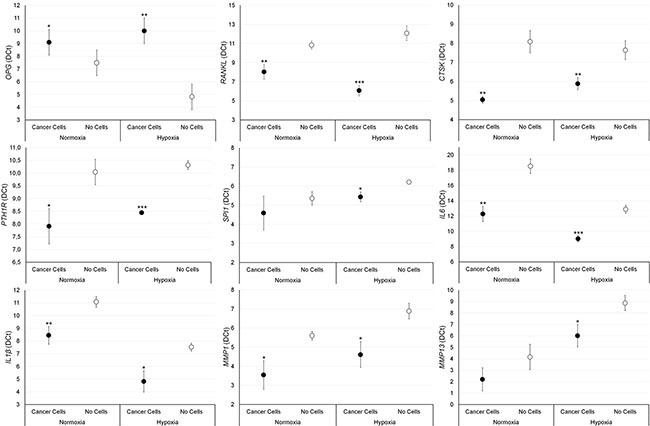
Gene expression analysis (*n* = 3 replicates) in female bone fragments cultured with or without MCF-7 cancer cells, both in normoxic and hypoxic conditions RANKL (normoxia: **p < 0.005; hypoxia: ***p < 0.0005), CTKS (normoxia and hypoxia: **p < 0.0005), PTH1R (normoxia: *p < 0.05; hypoxia: ***p < 0.0005), IL-6 (normoxia: **p < 0.005; hypoxia: ***p < 0.0005), IL-1β (normoxia: **p < 0.005; hypoxia: *p < 0.05), MMP-1 (normoxia and hypoxia: *p < 0.05), SPI1 (hypoxia: *p < 0.05) and MMP-13 (hypoxia: *p < 0.05): bone fragments with MCF-7 *versus* bone fragments without cancer cells. OPG (normoxia: *p < 0.05; hypoxia: **p < 0.005): bone fragments without cancer cells *versus* bone fragments with MCF-7.

**Figure 6:**
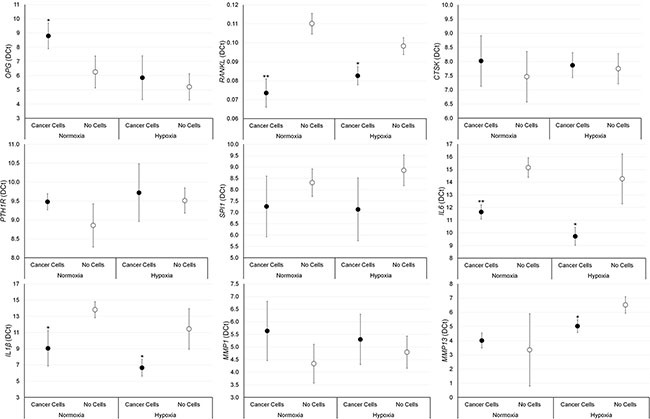
Gene expression analysis (*n* = 3 replicates) in male bone fragments cultured with or without PC-3 cancer cells, both in normoxic and hypoxic conditions RANKL (normoxia: **p < 0.005; hypoxia: *p < 0.05), IL-6 (normoxia: **p < 0.005; hypoxia: *p < 0.05), IL-1β and (normoxia and hypoxia: *p < 0.05) MMP-13 (hypoxia*p < 0.05): bone fragments with PC-3 versus bone fragments without cancer cells. OPG (normoxia and hypoxia: *p < 0.05): bone fragments without cancer cells versus bone fragments with PC-3.

The effect of breast and prostate cancer cell on bone fragments was investigated through gene expression analyses. Results are summarized in Figures 5 and 6. Significantly higher values of RANKL, CTKS, PTH1R, IL-6, IL-1β and MMP-1 were found in bone fragments cultured with MCF-7 breast cancer cells in comparison to bone fragments cultured without cancer cells, both in normoxic and hypoxic conditions. Conversely, SPI1 and MMP-13 showed significantly higher values only under hypoxic conditions (Figure 5). Bone fragments cultured with MCF-7 breast cancer cells revealed significantly lower OPG expression in comparison to bone fragments cultured without MCF-7 (Figure 5). Bone fragments cultured with PC-3 prostate cancer cells revealed a significantly higher gene expression for RANKL, IL-6 and IL-1β compared to bone fragments cultured without cancer cells, both in normoxic and hypoxic conditions, while MMP-13 showed significantly higher values only in hypoxic condition (Figure 6). Significantly lower OPG gene expression was observed in bone fragments cultured with PC-3 compared to bone fragment cultured without cells, but only in normoxic condition (Figure 6).

### Histological and immunohistochemical analyses

**Figure 7:**
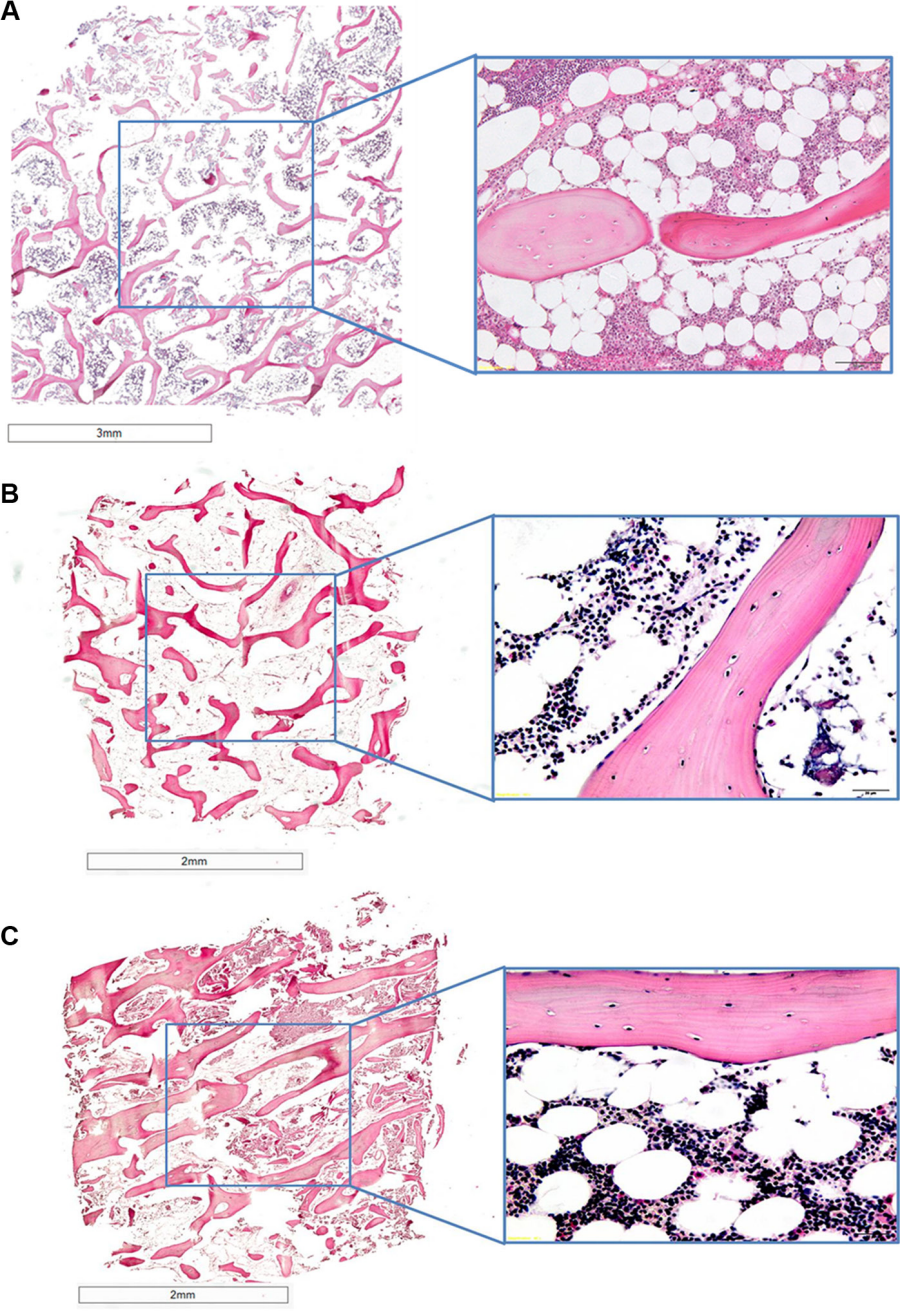
H/ E stained sections of human bones grown in the absence of cancer cells at (**A**) T0 and (**B**) and (**C**) at T1, (B) in normoxic conditions and (C) hypoxic conditions.1.25× and 40× of magnification

Femoral head specimens without cancer cells at T0 revealed typical trabecular bone architecture consisting of bone spicules (with osteocytes and osteoblasts) throughout the marrow compartment (Figure 7A). Marrow cells including hematopoietic (red and white blood cell progenitors) and stromal cells (with numerous adipocytes) were present within the bone spicules (Figure 7A).

After 7 days of culture in hypoxic and normoxic conditions (Figure 7B and 7C) bone specimens maintained the same architecture and morphology as those at T0 (Figure 7A). In fact, bone trabeculae presented numerous osteocytes in lacunae and osteoblasts strictly associated to bone spicules (Figure 7B and 7C). As for bone fragments at T0 the presence of marrow cells and stromal cells with many adipocytes within the bone spicules was observed (Figure 7B and 7C).

**Figure 8:**
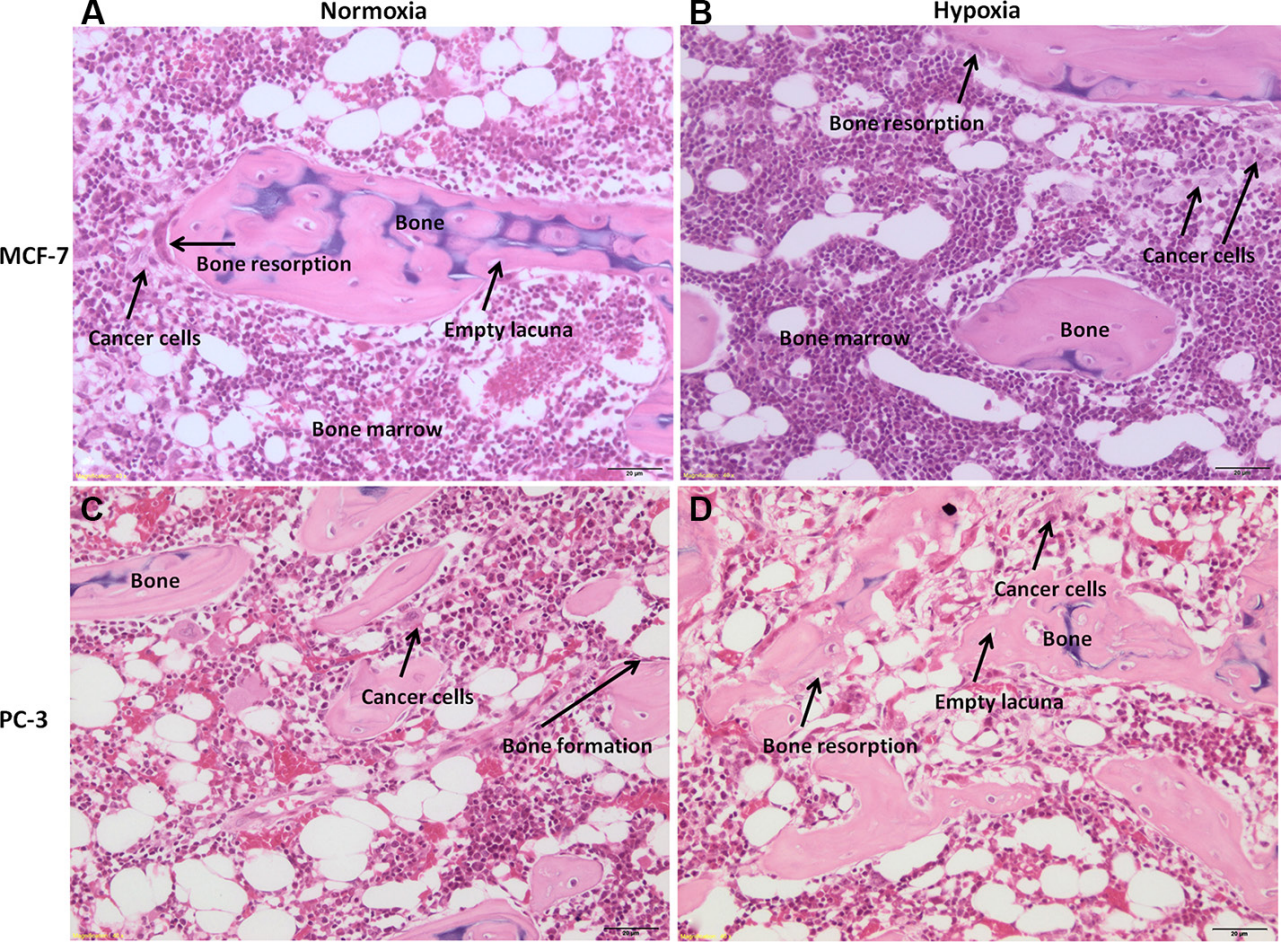
H/ E stained sections of human bone femoral head specimens grown in the presence of human cancer cells, both MCF-7 or PC-3, under normoxic and hypoxic conditions (**A** and **B**) Human bone from females patients cultured in the presence of MCF-7 breast cancer cell lines under (A) normoxic and (B) hypoxic conditions. (**C** and **D**) Human bone from males patients cultured in the presence of PC-3 prostate cancer cell lines under (C) normoxic and (D) hypoxic conditions. All sections are at 40× magnification.

Bone specimens cultured with cancer cells, MCF-7 or PC-3, under normoxic or hypoxic conditions, revealed an altered bone architecture and morphology compared to specimens cultured without cancer cells (Figure 8). Bone trabeculae were locally surrounded by malignant cells with tubule formations or solid cell clusters (Figure 8). Bone surface was at different phases of bone remodeling or different phases of bone resorption with, consequently, various morphological characteristics of the resorption lacunae. In addition, bone cell reaction varied from case to case. Morphological changes related to bone resorption included lacunar osteolysis and an initial trabecular fragmentation (Figure 8B and 8D). Additionally, in some cases osteocytes in the trabecular bone had disappeared leaving empty lacunae (Figure 8A and 10D). The altered architecture of trabecular bone may be ascribed to tumor cell and osteoclast activity as highlighted by the presence of osteoclasts along the trabeculae in bone specimens cultured with MCF-7 or PC-3 (Figure 8). In addition, infiltration in bone marrow spaces by malignant tumour, typically composed of foci of neoplastic cells with two or more hyperchromatic nuclei per cell, were observed in the specimens cultured with cancer cells, both MCF-7 and PC-3, under normoxic and hypoxic conditions (Figure 8). A histological analysis of the degree of bone resorption areas showed distinctions between different cancer cell types, MCF-7 and PC-3. In fact, in the specimens cultured with PC-3, both under normoxic and hypoxic conditions, an initial osteoblastic activity with non-lamellar (fibrous-like) bone tissue formation, with presence of osteoblasts along the pre-existing trabecule, was also seen (Figure 8C and 8D).

**Figure 9:**
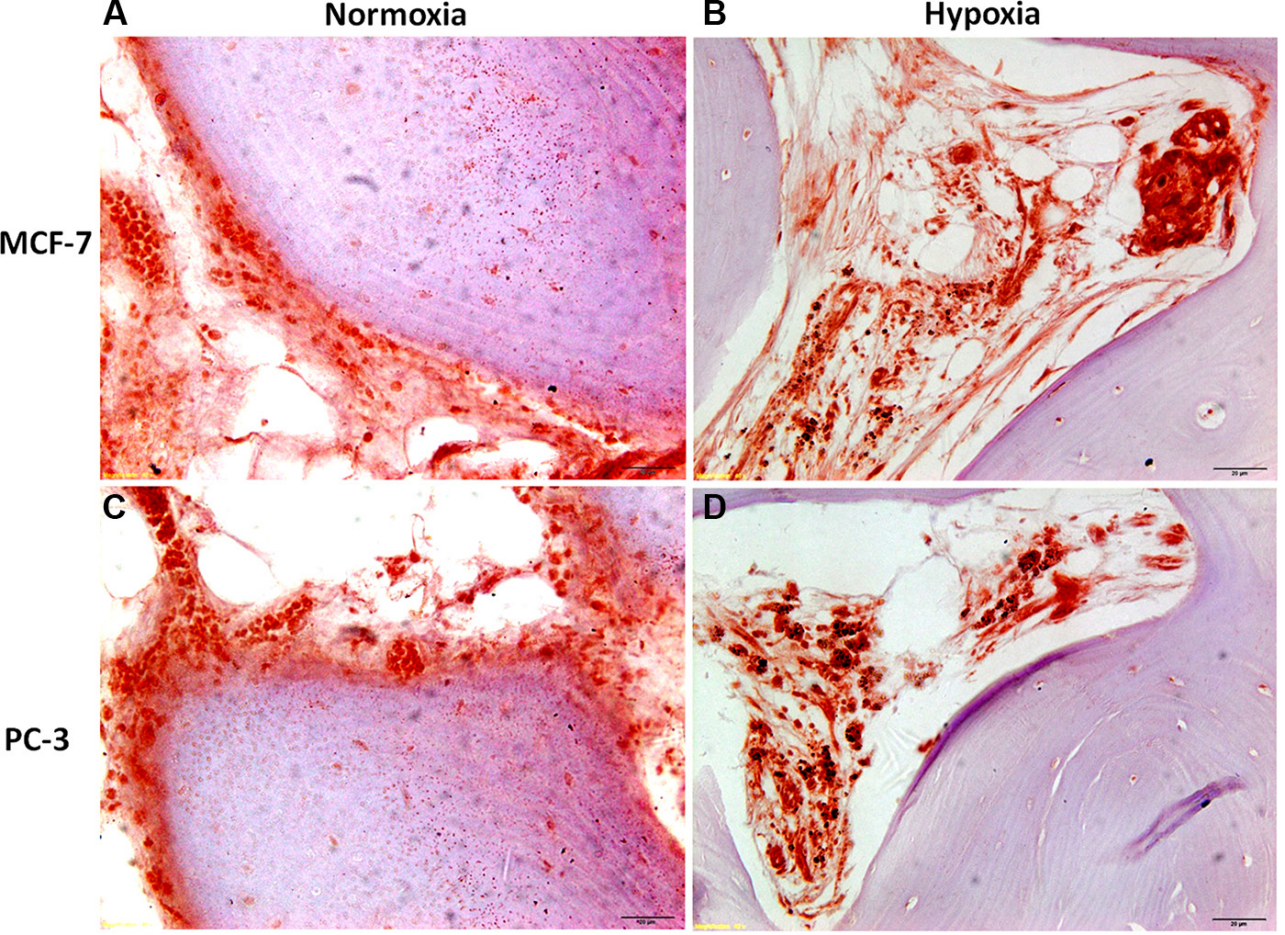
Immunohistochemical staining of femoral head bone specimens after co-culture with (**A** and **B**) MCF-7 and (**C** and **D**) PC-3 cells for 7 days with cytokeratin 18 antibody reveals (A) and (B) MCF-7 breast cancer cell and (C) and (D) prostate cancer cell colonization (in red) of the bone marrow compartment and attachment to ossified bone surface. All sections are at 80× magnification.

**Figure 10:**
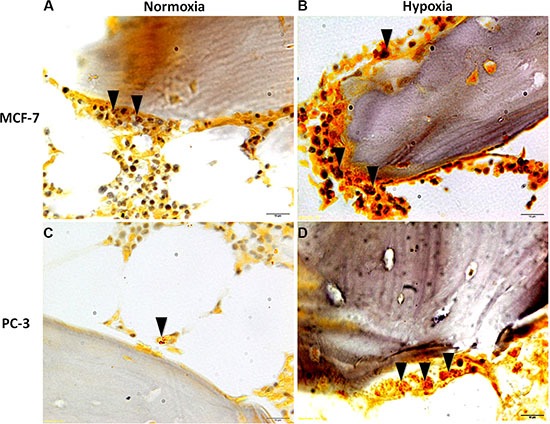
TRAP staining of femoral head bone specimens after co-culture with (**A** and **B**) MCF-7 and (**C** and **D**) PC-3 cells for 7 days. Presence of differentiated multinucleated osteoclasts throughout the trabecular bone (black arrows). All sections are at 80× magnification.

Post co-culture immunohistochemical staining with anticytokeratin 8 and 18 antibody of bone specimens with MCF-7 or PC-3, under normoxic and hypoxic conditions at 7 days, confirmed the histological results showing infiltration of tumor cells into the marrow compartment, predominantly in hypoxic condition, (Figure 9) and onto ossified bone surfaces.

Finally, TRAP staining after 7 days of culture with MCF-7 or with PC-3, both in hypoxic and normoxic conditions, revealed the presence of differentiated multinucleated osteoclasts throughout the trabecular bone (Figure 10A and 10B). However, a higher number of multinucleated active osteoclasts were observed in the specimens cultured with MCF-7 in comparison to those cultured with PC-3. Furthermore, the presence of a greater amount of differentiated multinucleated active osteoclasts was detected in the hypoxic condition.

## DISCUSSION

Currently, with the growing acceptance of *in vitro* models as effective tools for studying cancer biology, many enhanced and novel 3D models have emerged in an effort to recapitulate the native tumor and its microenvironment [17–25]. We developed a 3D model of bone metastases using human bone tissue taken from total hip replacement surgery patients cultured with human breast or prostate cancer cells (MCF-7 and PC-3) and established a *“proof of concept”* to recapitulate the bone metastasis and its microenvironment.

The main reason for the development of this dynamic 3D *in vitro* model was the need for a suitable model which would take into account the critical importance of the species-specific osteotropism that is essential in the study of bone metastases. The development of this model has a direct relevance in the study of bone metastases because it mimics more closely metastatic microenvironments in humans and provides a compromise between the reductionist approach which isolates cancer cells as a 2D monolayer, and the manufactured complexity of growing human tumors in xenogeneic hosts. Additionally, a model that so faithfully reproduces human metastases might be important to evaluate new therapeutic interventions to prevent and treat bone metastases.

The 3D cancer-bone metastasis model devised in this study was developed using a rolling apparatus system, where human breast or prostate cancer cells in suspension were cultured with free floating female and male human bone fragments, isolated from discarded total hip replacement surgery. The use of the rolling apparatus exposes equally all the surfaces of the human bone fragments to the cancer cells. In addition, to reflect the *in vivo* conditions we also took into account the hypoxic condition to mimic the nutrient and oxygen insufficiency at the tumor-host interaction. In fact, bone is a hypoxic microenvironment (pO_2_ between 1–7%) [26], which increases the growth of metastatic tumor cells that have adapted for survival in conditions of low O_2_.

We studied cancer/cell bone interaction over a relatively long period of up to 7 days. Unlike other reports [18, 25] we did not observe a decline in bone marrow viability after 7 days (T1) of culture as revealed by the Alamar Blue test and appearance in histological sections. In fact, the bone viability results obtained immediately after surgery (T0) were maintained after 7 days (T1) of culture with and without cancer cells (MCF-7 and PC-3), in both normoxic and hypoxic conditions.

Co-culture of human bone fragments with human breast or prostate cancer cells in suspension were evaluated for protein expression level and for gene expression profile. Co-culture of bone with MCF-7 cells, both in hypoxic and in normoxic conditions, resulted in increased transcription of genes associated with osteoclasts division, differentiation, and activation, such as RANKL, CTSK, PTH1R, IL-6 and IL-1β while there was a repression of OPG, which is associated with osteoblasts maturation [27–30]. The up-regulation of RANKL, IL-1 β but also TNF-α and the down-regulation of OPG have also been observed at protein expression level. This increased transcription of genes and protein expression, associated with the activation and function of osteoclasts, was further emphasized under hypoxic condition where an increased transcriptional activity of SPI1, a gene involved in a transcriptional regulatory network typically associated with osteoclastogenesis, was observed [31–33]. In addition to destructive bone loss and related clinical complications, tumor-induced osteoclasts contribute also to the establishment, growth, and survival of tumors. In fact, products of osteoclastic bone resorption increase tumor cell proliferation and survival and cause further production of osteolytic and osteoblastic factors, thereby creating a positive feedback loop known as the “vicious cycle”. As osteoclasts are highly related to macrophages, it is well known that many pro-tumorigenic factors can be produced by osteoclasts directly, including IL-1, TNF and IL-6, in addition to matrix metalloproteinases [34–35]. MMPs are also important mediators of bone resorption, yet only have the capacity to breakdown proteins in non-mineralized bone [27]. The main MMPs associated with bone resorption are MMP-1, MMP-9, and MMP-13; all of which are collagenases [36]. High levels of MMPs have also been related to the metastatic characteristics of a number of tumors, and in our model we found an increased transcriptional activity of MMP-1 and MMP-13, the latter only in hypoxic environment. These data closely reflects the osteolytic characteristic of MCF-7 breast cancer cells and therefore this model has also the ability to correlate the specific metastatic potential of this cells on bone, in particular under hypoxic conditions. In fact, hypoxia stimulates blood cell proliferation and blood vessel formation, and modulates the expression of extracellular matrix components and remodeling enzymes, thereby maintaining tissue homeostasis. In contrast to the strong osteolytic effects on human bone fragments co-cultured with MCF-7 cells, when PC-3 prostate cancer cells were co-cultured with human bone we were able to show that there was an initial subtle mixed effect (i.e., osteolytic and osteoblastic). Co-culture of bone with PC-3 cells, both in hypoxic and in normoxic conditions, resulted in increased transcription of RANKL, IL-6 and IL-1β genes and in a repression of OPG gene expression. However, no differences were seen concerning CTSK, PTH1R and SPI1 expression that are key genes in the transcriptional regulatory network of osteoclastogenesis. In addition, different results were obtained for protein expression level where no significant change in RANKL expression and higher levels of OPG, only in normoxic condition, were found. We hypothesized that this different gene and protein expression was associated to the fact that OPG may play an autoregulatory role during osteoclastogenesis in an intrinsic mechanism that negatively regulate its expression. A negative feedback in osteoclastogenesis was already reported by Kang et al. [37] who suggested that OPG, expressed by the osteoclasts themselves, may play an auto-regulatory role during osteoclastogenesis through the induction of apoptosis. However, despite our hypothesis on the possible autoregulatory role of OPG during osteoclastogenesis, this study underlined the presence of still numerous doubts regarding the serious question of how the OPG/RANKL/RANK or OPG/TRAIL/death receptor system interacts during osteoclastogenesis. Finally, it should be carefully noted that the correlation between gene expression and protein levels has always been a very intriguing issue. Interpreting protein levels based on mRNA expression may be misleading. In our experience, protein levels do not necessarily reflect gene expression levels. In fact, change in mRNA levels and protein levels may not correlate well mainly due to the regulation control at different levels, i.e transcriptional regulation and post-transcriptional regulation (RNA processing, RNA stability, translational, protein stability and protein modification). Concerning TNF-α, that enhances osteoclasts function leading to bone degradation, its up-regulation, also observed at the protein expression level. Finally, also an increase in the transcriptional activity of MMP-13, only under hypoxic condition, was observed when PC-3 cells were present. MMP-13 is an important mediator of cell-tumor communications by processing soluble factors, such as RANKL, which in turn stimulate osteoclastogenesis [27]. In addition, MMP-13 is implicated in the enzymatic cascade because proMMP-13 is activated by MMP-2 and by MMP-14, which can also turn on proMMP-2. Once active, both MMP-2 and MMP-13 take part in the activation of proMMP-9 into MMP-9. In this way, MMP-13 is implicated both in metastatic and non-metastatic tumors, where molecular expression is spurred by numerous cytokines, growth factors and tumor promoters that act on tumor cells.

Results of protein expression level and of gene expression profile were confirmed also by histology; co-culture with human breast or prostate cancer cells in suspension with free-floating human bone fragments caused local trabecular bone architectural alteration. In addition, marked cancer cell penetration with characteristic foci of neoplastic cells, with two or more hyperchromatic nuclei for cell, was observed in bone fragments cultured both with breast or prostatic cancer cells. MCF-7 and PC-3 cells migrated towards the femoral head bone specimens and also into the bone marrow spaces. The results also denoted different degrees of bone resorption areas between the different cancer cell types (MCF-7 or PC-3). In fact, bone metastases from MCF-7 cancer cells were nearly always associated with osteolysis, whereas bone metastases from PC-3 cancer cells were prevalently mixed (i.e., osteolytic and osteoblastic) [22]. Thus, our model reflects closely the osteolytic and the osteolytic/osteoblastic (mixed type) characteristics of the cancer cell lines used and hence the model also has the ability to correlate the specific metastatic potential of these cells on bone. Cancer cell colonization of bone tissue was also confirmed with immunohistochemical analyses, which revealed cytokeratin positive breast and prostate cancer cells within the marrow and ossified compartments. In hypoxic conditions both MCF-7 and PC-3 colonized mainly the bone marrow compartment. This is probably due to the bone marrow environment, which in hypoxic conditions is more susceptible to the invasion and growth of tumor cells [38–39]. Additionally, a greater amount of multinucleated osteoclasts were observed in the hypoxic condition with respect to that of the normoxic one. The ability of the cancer cells to promote the formation of active osteoclasts is a special property of tumors which metastasize to bone, and a necessary requirement to initiate and sustain tumor expansion. Sabino et al. [40] showed that under hypoxic conditions tumor cells produce prostaglandin E2 (PGE2) at increased levels. PGE2 is synthesized in bone principally by osteoblasts to stimulate bone resorption and is regulated by cyclo-oxygenase (COX) enzymes. Miyaura et al [41] observed that in cancer metastases and bone resorption PGE2 binds to the EP4 receptor (a PGE receptor subtype) to induce RANKL expression and stimulation of bone destruction.

This model was also evaluated by a 4D computational methodology by micro-CT to track the presence of changes in bone volume fraction, BMD and TMD in a non-invasive simultaneous manner as a result of the presence of cancer cells. This 4D approach has several strengths, as it allows, besides achieving 3D measures similar to traditional 2D histology, for a temporal characterization of the occurring bone (re)modeling sequences. Thus, this computational methodology provides several new ways to examine bone turnover, including the temporal character of formation and resorption processes and (re)modeling sequences both in healthy and pathological conditions. In this application, after recording micro-CT datasets of the femoral head bone fragments at T0 without cells and at T1 after 7 days of culture with cancer cells, the 2 volumes were superimposed, thus allowing the measurement of bone remodeling and mineral density over time. After 7 days of culture, no significant differences were found among bone specimens both in hypoxic and in normoxic conditions. However, in normoxic condition we found that BMD values increased in bone cultured with MCF-7 and decreased in PC-3 cultured bone. Moreover, in hypoxic conditions TMD decreased in bone femoral head specimens cultured with both cancer cells in comparison to the same specimens at T0 in hypoxic conditions. These result highlighted how hypoxia can promote a more physiological environment, which leads to bone resorption in breast cancer metastases, and it induces more bone resorption that bone formation in prostate cancer metastases. The lower BMD and TMD found in this study indicated a decrease in bone mineral content that is the primary recognizable cause of bone loss and subsequent osteolysis [42]. Bone density measurement is thought to be the most reliable and reproducible method for assessment and quantification of bone metastases in different anatomical districts [43].

Promising results were thus obtained from this 3D model; however some limitations of the study and a future possible upgrade should be considered by using a larger sample. Firstly, we followed cell/bone interaction up to 7 days, thus an essential improvement would be to optimize culture conditions for longer experimental times also in order to study microstructural and density bone parameters. Secondly, further exploration of any molecular change should be performed in order to identify other key pathways and factors that might contribute to bone metastases. Finally, the lack of a functioning circulatory system in this model prevents the study of cancer cell extravasation.

In conclusion, this dynamic 3D system supports the *“proof of concept”* for the application of this model for the recapitulation of *in vivo* cancer-bone metastasis spread, in particular monitoring and controlling hypoxia that seems to better mimic physiological tumors condition. Moreover, the model might serve as an efficient system at a higher level than the 2D cell culture model, but at a lower ethical cost. The proposed system, in comparison to other models, seems to be cost effective and consequently a greater amount of experiments might be performed to obtain extensive datasets for reliability, reproducibility and statistical analysis.

The versatility of this 3D model offers the possibility to further explore the application of the model for other clinical applications, e.g. increasing the biological complexity of the system by adding other cell types or increasing the culture time. This will ultimately benefit the development of new therapies and improve disease management. In fact, in the future, we plan to use this model also for characterizing other metastatic cancer cells from patients, thus highlighting the physiological events that occur when cancer cells encounter the bone. All these aspects will greatly enrich the existing knowledge on the bone metastasis by providing a specific link to the clinical situation, thus making this 3D model an attractive tool for multidisciplinary experts, namely clinicians, biologists and bioengineers.

## MATERIALS AND METHODS

### Human bone tissue cultures

The study was approved by the Ethics Committee of the Rizzoli Orthopedic Institute (Protocol MET-3D; approved May 22, 2014) and informed consent was obtained from all subjects. The main clinical characteristics of the patients are listed in Table 1. Exclusion criteria were human immunodeficiency virus (HIV), hepatitis B virus (HBV), hepatitis C virus (HCV), pregnancy, osteoporosis, primary bone tumors and bone metastases, minors and/or patients incapable of giving consent personally.

**Table 1:** Clinical features of patients undergoing total hip replacement

	Patient 1.	Patient 2.	Patient 3.	Patient 4.	Patient 5.	Patient 6.	Patient 7.	Patient 8.	Patient 9.	Patient 10.	Patient 11.	Patient. 12
**Gender**	Female	Female	Female	Female	Female	Female	Male	Male	Male	Male	Male	Male
**Age**	60	68	58	60	75	61	79	82	60	61	58	73
**Height (cm)**	155 cm	158 cm	153 cm	162 cm	157cm	146 cm	160 cm	160 cm	181 cm	170 cm	157 cm	175 cm
**Weight (Kg)**	75 Kg	85 Kg	60 Kg	60 kg	58 Kg	71 Kg	72 kg	58 Kg	84 Kg	82 Kg	84 Kg	60 Kg
**BMI**	31.22 Kg/m^2^	34.05 Kg/m^2^	25.63 Kg/m^2^	22.9 Kg/m^2^	23.5 Kg/m^2^	33.8 Kg/m^2^	28.12 Kg/m^2^	22.66 Kg/m^2^	25.64 Kg/m^2^	28.40 Kg/m^2^	34.10 Kg/m^2^	19.00 Kg/m^2^
**BMD**	≥ −1.0	≥ −1.0	≥ −1.0	≥ −1.0	≥ −1.0	≥ −1.0	≥ −1.0	≥ −1.0	≥ −1.0	≥ −1.0	≥ −1.0	≥ −1.0
**Comorbidities**	No	Hypertension, hypothyroidism, depression, gastritis, ischemic heart disease.	No	Depression, heart disease.	Depression.	Hypertension	No	Prostatic hypertrophy, chronic obstructive pulmonary disease, heart disease.	Hypertension	No	Hypertension, hypercholeste rolemia	Hypertension, diabetes
**Use of drugs active on bone metabolism**	No	No	No	No	No	No	No	No	No	No	No	No

**Figure 11:**
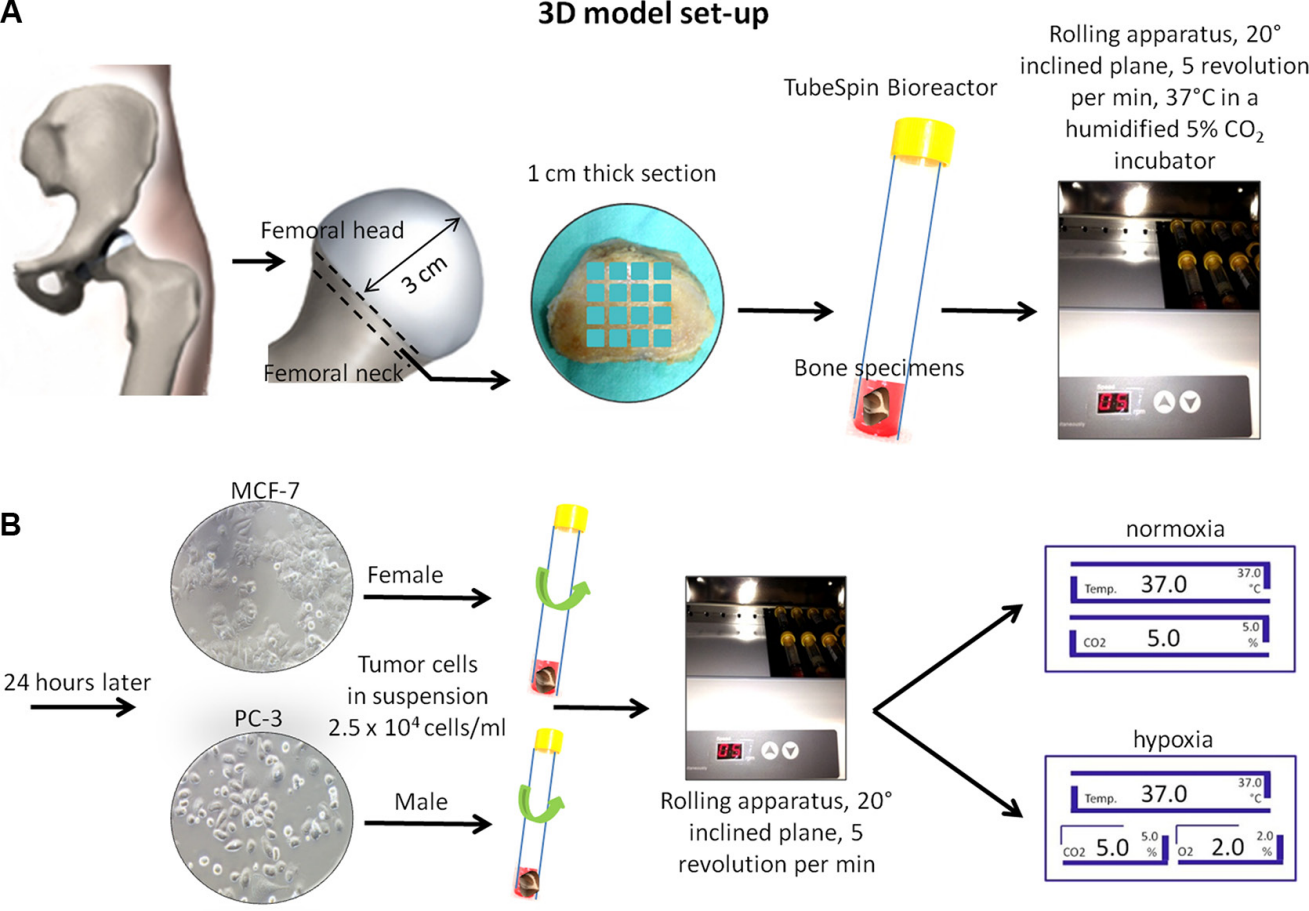
Schematic representation of the 3D model set-up (**A**) Femoral head and neck resection and cut of 1-cm thick section. Bone femoral samples (1 × 1 cm) were placed in a TubeSpin Bioreactor and with a 20° inclined plane in a rolling apparatus at 5 revolutions per minute for 24 hours at 37°C in a humidified 5% CO_2_ incubator. (**B**) After 24 hours 2.5 × 10^4^ cells/ml in suspension (MCF-7 in female bone specimens and PC-3 in male bone specimens) were added. Rolling TubeSpin were cultured for 7 days in a rolling tube apparatus (20° inclined plane and 5 revolutions/min) at 37°C in hypoxic conditions: in a humidified 2% O_2_, 5% CO_2_ and 88% N_2_ incubator or in normoxic conditions: in a humidified 5% CO_2_ incubator.

Femoral heads were collected from twelve patients, 6 female and 6 male undergoing total hip replacement at Rizzoli Orthopedic Institute. Briefly, with the patient in supine position a minimally invasive direct lateral approach was performed. After anterior capsulotomy the hip was dislocated anteriorly and a femoral neck osteotomy was performed approximately one cm proximally to the lesser trochanter. Subsequently, at a distance of about 3 cm from the articular cartilage, a 1-cm thick section of the same width as the femur was cut with a surgical saw. From this section we obtained 16 bone specimens *per* patient with height and length equal to 1.0 ± 0.3 cm and weighing between 0.8 and 1.0 g. Bone specimens were placed singularly in a 15 ml TubeSpin Bioreactor, where the gas exchange is ensured by a screw cap with a 0.22 μm filter membrane (TPP TubeSpin^®^ Bioreactors, TPP Techno Plastic Products, Switzerland), containing 3 ml of culture medium. The live organ culture medium consisted of Dulbecco's Modified Eagle's Medium (DMEM, Sigma-Aldrich, MO, USA) supplemented with 5 mg/ml of bovine serum albumine (BSA, Sigma-Aldrich, MO, USA), 2 mM glutamine and antibiotics (100 U/ml penicillin, 100 μg/ml streptomycin) (Gibco, INVITROGEN Corporation, Carlsbad, CA) and with no fetal calf serum. The bone specimens in the TubeSpin Bioreactor, were transported to the laboratory within 20 minutes after surgery and twelve of them were immediately placed in a rolling apparatus (Thermo Scientific, Waltham, MA, USA) with a 20° inclined plane at 5 revolutions per minute for 24 hours at 37°C in a humidified 5% CO_2_ incubator (Thermo Scientific, Waltham, MA, USA) (Figure 11A). Two specimens were used as positive controls and immediately evaluated for viability by the alamar blue test. The remaining two bone specimens were used as negative controls, they were soaked for five times in liquid nitrogen (−196°C) for 4 min and then incubated (37°C) for 4 min to induce total necrosis of the trabeculae and bone marrow depletion.

### Cells lines and cell culture

The human breast adenocarcinoma MCF-7 cell line was cultured using DMEM medium (Sigma-Aldrich, MO, USA) whereas the human prostate cancer PC-3 cell line was cultured in RPMI 1640 (GIBCO, Invitrogen, Life Technologies, CA, USA) at 37°C in a humidified 5% CO_2_ incubator (Hera Cell 150i; Thermo Scientific, Waltham, MA, USA). Mediums contained 10% FCS (Lonza, Verviers, Belgium) and 2 mM glutamine and antibiotics (100 U/ml penicillin, 100 μg/ml streptomycin) (Gibco, INVITROGEN Corporation, Carlsbad, CA). Cultures were supplemented with fresh medium every two days.

### 3D co-culture system

After 24 hours at 37°C in a humidified 5% CO_2_ incubator on a rolling apparatus, 2.5 × 10^4^ cancer cells/ml in suspension (MCF-7 in female bone specimens and PC-3 in male bone specimens) were added to six of the twelve bone specimens cultured in the TubeSpin Bioreactor. Three of them were cultured for a further 7 days in a rolling apparatus with a 20° inclined plane at 5 revolutions per min at 37°C in hypoxic conditions in a humidified 2% O_2_, 5% CO_2_ and 88% N_2_ incubator (Hera Cell 150i; Thermo Scientific, Waltham, MA, USA), whereas the other three tubes were cultured in normoxic conditions: in a humidified 5% CO_2_ incubator (Hera Cell 150i; Thermo Scientific, Waltham, MA, USA) (Figure 11A and 11B). The remaining six bone specimens were cultured without cancer cells, both in hypoxic (*n* = 3) and in normoxic (*n* = 3) conditions.

After 7 days of culture bone specimens from 6 patients, 3 female and 3 male, were immediately processed for bone viability, densitometric and structural evaluations by micro-CT analyses and successively fixed as described above for histological and immunohistochemical analyses. Bone specimens from the remaining 6 patients, 3 female and 3 male, were used for gene expression analyses. Media were collected, centrifuged and stored at −20°C for ELISA protein assays.

Finally, six TubeSpin Bioreactor tubes with breast cancer cells alone, MCF-7, and 6 for prostate cancer cells, PC-3, (2.5 × 10^4^ cells/ml) were incubated at 37°C for 7 days on the rolling apparatus (20° inclined plane and 5 revolution/min) in hypoxic and in normoxic conditions.

The summary of the experimental set up is shown in Table 2.

**Table 2:** Summary of the experimental set-up

Experimental set-up	
Two femoral head specimens	Immediately evaluated for viability and gene expression analyses
Two femoral head specimens	Immediately used to induce total necrosis of the trabeculae and bone marrow depletion and evaluated for viability and gene expression analyses as control
Three femoral head specimens in the TubeSpin Bioreactors	Cultured with 2.5 × 10^4^ cancer cells/ml for 7 days in normoxic conditions and evaluated for viability, ELISA protein assay, densitometric and structural analyses, gene expression analyses and for histological and immunohistochemical tests
Three femoral head specimens in the TubeSpin Bioreactors	Cultured with 2.5 × 10^4^ cancer cells/ml for 7 days in hypoxic conditions and evaluated for viability, ELISA protein assay, densitometric and structural analyses, gene expression analyses and for histological and immunohistochemical tests
Three femoral head specimens in the TubeSpin Bioreactors	Cultured for 7 days in normoxic conditions and evaluated for viability, ELISA protein assay, densitometric and structural analyses, gene expression analyses and for histological and immunohistochemical tests
Three femoral head specimens in the TubeSpin Bioreactors	Cultured for 7 days in hypoxic conditions and evaluated for viability, ELISA protein assay, densitometric and structural analyses, gene expression analyses and for histological and immunohistochemical tests
2.5 × 10^4^ cells/ml of MCF-7 or PC-3 in the TubeSpin Bioreactors	Cultured for 7 days in normoxic conditions and evaluated for viability, ELISA protein assay and gene expression analyses
2.5 × 10^4^ cells/ml of MCF-7 or PC-3 in the TubeSpin Bioreactors	Cultured for 7 days in hypoxic conditions evaluated for viability, ELISA protein assay and gene expression analyses

### ELISA protein assay

Supernatants from each culture condition were collected and centrifuged to remove particulates. Aliquots were dispensed in Eppendorf tubes for storage at −20°C and assayed for osteoprotegerin (OPG), Receptor Activator for Nuclear factor KB Ligand (RANKL), interleukin 1β (IL-1β), and tumor necrosis factor α (TNF-α) (Boster Biological Technology Co. Ltd, Wuhan, China). The concentration of each factor (OPG, RANKL, IL-1β and TNF-α) was normalized by the weight of the bone fragment.

### Bone tissue viability

The alamar blue test (Serotec, Oxford, UK) was used to evaluate viability of bone at time 0 (T0) (both in positive controls and in negative controls) and after 7 days of culture (T1). The reagent is a dye, which incorporates an oxidation-reduction (REDOX) indicator that changes color in response to the chemical reduction of growth medium, resulting from cell growth. It was added to each bone specimen (1:10 v/v) for 4 h at 37°C. After transferring the supernatants to 96-well plates, the absorbance of supernatant was read spectrophotometrically at 570 and 600 nm wavelengths (for the fully oxidized and reduced forms of reagent) by a microplate reader (BioRad, CA, USA). The results, obtained as optical density (OD) data, were processed following the manufacturer's instructions and expressed as reduction percentage.

### 4D micro-CT analyses

**Figure 12:**
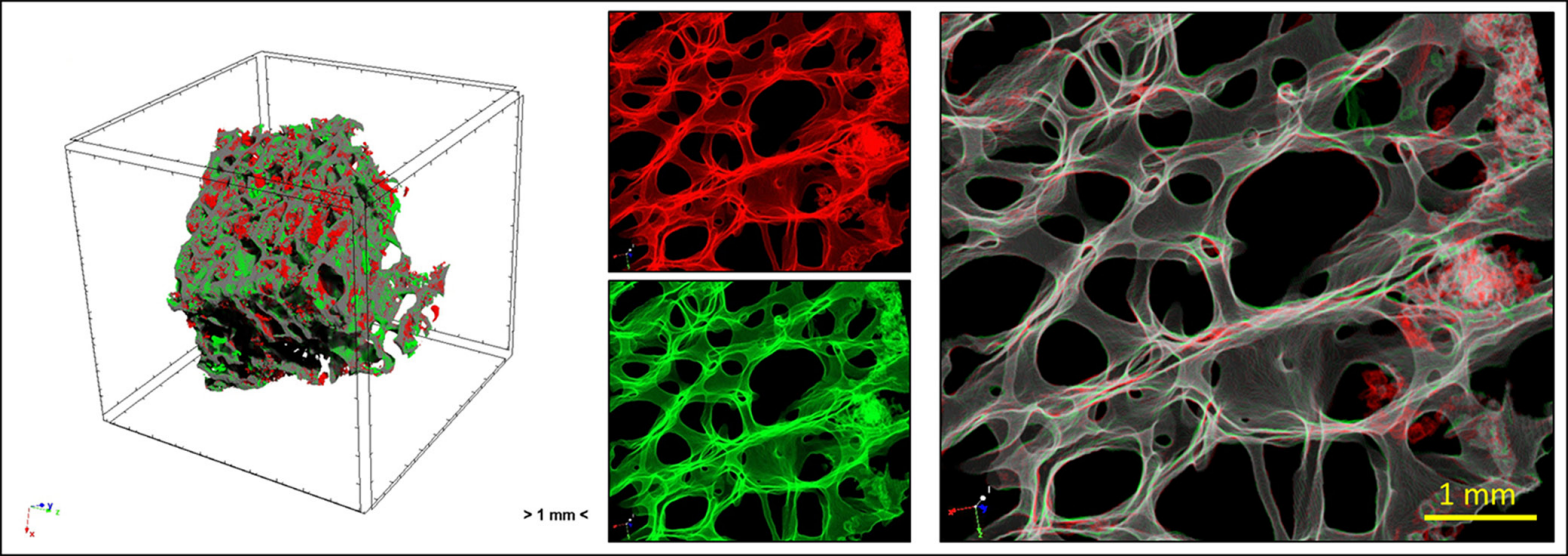
Overview of the study by micro-CT analysis: human femoral head bone specimens were scanned at T0 and T1 and 4D micro-CT analyses was applied to identify regions of bone formation (green) and resorption (red)

After bone viability assessment femoral head bone specimens were scanned both at the beginning and at the end of the 7 days' cell culture using the Skyscan 1172 micro-CT system (Bruker microCT, Kontich, Belgium). The specimens were scanned in their culture medium at 70 kV of source voltage and 140 μA, with a total rotation of 180° and a rotation step of 0.4°. An aluminum filter of 0.5 mm was used between the source and the sample. The image pixel size was 12 μm and the scan duration was nearly 45 minutes for every specimen (software Skyscan 1172 version 1.5 build 14, Bruker microCT, Kontich, Belgium). The reconstructions were performed using the software NRecon (version 1.6.9.16, Bruker micro-CT, Kontich, Belgium) and the resulting jpg images had a resolution of 2000 × 2000 pixels with a pixel size of 12 μm. Beam hardening, ring artifacts and the specific misalignment corrections were used. The datasets of the samples before cell culture (reference dataset) and after 7 days (target dataset) were co-registered using an intensity-based method in Dataviewer software (Bruker microCT, Kontich, Belgium). More specifically, rigid transformation (x/y/z translations and 3D rotations) and matching criteria based on sum of square difference were used. To overcome the problem of mismatching error, due to the presence of debris at the outer bulk surface of the specimens, a Volume of interest (VOI) was considered consisting of a 3D erosion of 0.5 mm of the bulk specimen volume. To evaluate the bone remodeling occurring as a result of tumor cell/bone cell interaction in our *in vitro* model at the different conditions, resorbed bone was defined as all bone voxels that were present at T0 but absent at T1, whereas formed bone was defined as bone voxels that were present at T1 but absent at T0. This resulted in a map that indicated the spatial locations of bone formation and resorption sites (Figure 12). The resulting map of bone formation and resorption was then used to obtain the Bone Volume Fraction (BV/TV %) parameter, expressed as the ratio between the volume of bone within VOI and the total volume of the VOI was evaluated. Moreover, Bone Mineral Density (BMD), inclusive porosity, and Tissue Mineral Density (TMD), exclusive porosity, were also calculated through calibration by two cylindrical phantoms with known concentrations of the mineral compound calcium hydroxyapatite (CaHA). The phantoms, with a diameter similar to that of the specimen (specifically 8 mm) and concentration of CaHA of 250 and 750 mg/cm^3^ were scanned with the same setting specification as that of the bone specimens.

### Gene expression analyses

After 7 days of culture, bone specimens were subjected to cryogenic grinding and pulverization, immersed in liquid nitrogen with Freezer/Mill^®^ 6770 (SPEX^®^ SamplePrep, Metuchen, NJ, USA), and total RNA extraction was performed by phenol/chlorophorm method withTrizol (Life Technologies, Thermo Scientific, Waltham, MA, USA). RNA was purified on columns with PureLink RNA Mini Kit (Life Technologies, Thermo Scientific, Waltham, MA, USA) according to the manufacturer's instructions. Total RNA was eluted with RNase-free water, quantified by NanoDrop 2000 (Thermo Scientific, Waltham, MA, USA) and the quality of RNA was evaluated by electrophoresis on 1% agarose gel. Each RNA sample (500 ng) was reverse transcribed to cDNA using the Super Script VILO cDNA Synthesis kit (Invitrogen, Life Technologies) according to the manufacturer's instructions and diluted to a final concentration of 5 ng/μl. Quantification of gene expression for 10 genes (Table 3) was performed in a LightCycler 2.0 Instrument (Roche Diagnostics GmbH, Mannheim, Germany) using QuantiTect SYBR Green PCR kit (Qiagen, Hilden, Germany). Primer details for all genes analyzed are reported in Table 3. Each sample was tested in duplicate. The protocol included:

– denaturation at 95°C for 15 min;
– 30 to 50 cycles of amplification (95°C 15 s, appropriate annealing temperature for each target gene and 72°C for 20 s);
– melting curve analysis to check for amplicon specificity.

**Table 3:** Primer details for the analyzed genes

Gene	Symbol	Primer Fw (5′ → 3′)	Primer Rv (5′ → 3′)	T annealing	Amplicon length	Target
Glyceraldehyde-3-phosphate dehydrogenase*	GAPDH	TGGTATCGTGGAAGGACTCA	GCAGGGATGATGTTCTGGA	56°C 20''	123 bp	NM_002046
Tumor Necrosis Factor (Ligand) Superfamily, Member 11 **	TNFSF11 (RANKL)	TGAGATGAGCAAAAGGCTGAG	AGGAGCTGTGCAAAAGGAAT	60°C 20''	134 bp	NM_033012
Osteoprotegerin **	TNFRSF11B (OPG)	CTACCAAGACACTAAGCCAGT	AAACAGTGAATCAACTCAAAAATGTG	60°C 20''	113 bp	NM_002546
Cathepsin K **	CTSK	CAGACAACAGATTTCCATCAGC	CTTCTTCCATAGCTCCCAGTG	60°C 20''	118 bp	NM_000396
Parathyroid hormone 1 receptor **	PTHR1	CTGCACAGCCTCATCTTCA	CTCTGACACTGACCCACAC	60°C 20''	115 bp	NM_000316
Hematopoietic Transcription Factor PU.1 **	SPI1	CAGGCGTGCAAAATGGAAG	GTAATGGTCGCTATGGCTCTC	60°C 20''	147 bp	NM_003120
Interleukin 6 **	IL6	GCAGATGAGTACAAAAGTCCTGA	TTCTGTGCCTGCAGCTTC	60°C 20''	120 bp	NM_000600
Interleukin 1betaI ***	IL1b	Hs_IL1B_1_SG		55°C 20''	117 bp	NM_000576
Matrix metallopeptidase 1 **	MMP1	GACAGAGATGAAGTCCGGTTT	GCCAAAGGAGCTGTAGATGTC	60°C 20''	102 bp	NM_001145938
Matrix metallopeptidase 13M **	MMP13	AGCCACTTTATGCTTCCTGA	TGGCATCAAGGGATAAGGAAG	60°C 20''	130 bp	NM_002427

* Designed with Primer Blast (http://www.ncbi.nlm.nih.gov/tools/primer-blast/).

** Prime Time assay IDT.

*** QuantiTect Primer Assay – Qiagen.

Data were collected using the LightCycler Software 4.1. Gene expression levels of the target genes were calculated by normalization to the reference gene GAPDH, using the comparative threshold method (ΔCt), so that the ΔCt value is lower the more the gene is expressed.

### Histological and immunohistochemical analyses

Bone tissue specimens were fixed in 10% buffered formalin and decalcified in a nitric/formic acid solution. When decalcification was complete (about 5–7 days), the samples were dehydrated in a graded series of alcohols and then processed for paraffin embedding. Five-micrometer-thick sections were obtained by a Microm HM340E (Microm International GmbH, Heidelberg, Germany) and stained with haematoxilin/eosin (H/E) or processed for immunohistochemical analysis using rabbit anti-human keratin 18 Monoclonal antibody (clone SP69) and rabbit anti-human keratin 8 polyclonal antibody (Spring Bioscience, Pleasanton, CA, USA). To reduce intra-bone variation, we evaluated at least three sections throughout the biopsy at 30 μm intervals. Ten microscopic fields per section (magnification 40× or 80×) were randomly observed with an optic microscope (Olympus-BX51, Germany) connected to an image analyzer system (Leica-Qwin, UK).

### TRAP staining to visualize osteoclast activity

Tissue sections were deparaffinised and TRAP staining was performed using a commercial acid phosphatase leucocyte kit (Sigma, St Louis, MO). In the bone samples, ten areas (magnification × 20) were randomly observed with an optic microscope (Olympus-BX51, Germany) connected to an image analyzer system (Leica-Qwin, UK) and TRAP positive cells in each area were observed.

### Statistical analysis

Statistical analysis was performed using the IBM^®^ SPSS^®^ Statistics v.23 software. Data are reported as Mean ± SD at a significance level of *p* < 0.05. The Kolmogorov Smirnov test was performed to test normality of the variables. The General Linear Model (GLM) with adjusted Sidak's multiple comparison test with ‘culture conditions’ (normoxia or hypoxia) and ‘presence of cancer cells’ (with or without) as fixed effects was performed to assess the differences between factors on micro-CT results within female and male bone specimens, separately. The differences between hypoxic and normoxic culture conditions in terms of cell viability were analyzed by using Student's *t-test* within female and male bone specimens, separately.
